# Pressurized IntraPeritoneal Aerosol Chemotherapy (PIPAC) for the treatment of malignant mesothelioma

**DOI:** 10.1186/s12885-018-4363-0

**Published:** 2018-04-18

**Authors:** Urs Giger-Pabst, Cédric Demtröder, Thomas A. Falkenstein, Mehdi Ouaissi, Thorsten O. Götze, Günther A. Rezniczek, Clemens B. Tempfer

**Affiliations:** 1 Basic Research Laboratories of the Department of Surgery, Marien Hospital Herne, Ruhr-Universität Bochum, Herne, Germany; 2 Department of General Surgery & Therapy Center for Peritoneal Carcinomatosis, Marien Hospital Herne, Ruhr-Universität Bochum, Herne, Germany; 3 Department of Digestive and Oncologic Surgery, Colorectal Surgery Unit, Trousseau Hospital, Tours, France; 4 Institute of Clinical Cancer Research, UCT-University Cancer Center Frankfurt, Hospital Northwest, Frankfurt, Germany; 5 Department of Obstetrics and Gynecology, Marien Hospital Herne, Ruhr-Universität Bochum, Herne, Germany

**Keywords:** PIPAC, PITAC, Peritoneal, Thoracal, Mesothelioma, Tumor regression

## Abstract

**Background:**

Patients with recurrent malignant epithelioid mesothelioma (MM) after surgery and standard chemotherapy with cisplatin and pemetrexed have limited treatment options.

**Methods:**

We performed a retrospective cohort study of patients with recurrent MM undergoing Pressurized IntraPeritoneal/Thoracal Aerosol Chemotherapy (PIPAC/PITAC) with doxorubicin 1.5 mg/m^2^ and cisplatin 7.5 mg/m^2^. Data were retrospectively collected in a prospective registry of patients undergoing PIPAC/PITAC. Study outcomes were microscopic tumor regression grade (TRG), survival and adverse events (v4.0 CTCAE).

**Results:**

A total of 29 patients (m/f = 17/12) with MM with a mean age of 62.4 (range: 42 to 84) years were analyzed. A total of 74 PIPAC and 5 PITAC procedures were performed. The mean number of PIPAC applications was 2.5 (range: 0 to 10) per patient. Twenty patients (69%) had > 2 PIPAC procedure and were eligible for TRG analysis. TRG 1 to 4 was observed in 75% (15/20) of patients. Major regression (TRG 3) or complete regression (TRG 4) was observed in 20% and 10%, respectively. PIPAC induced significant tumor regression in 51.7% (15/29) of patients with a cumulative effect after repetitive PIPACs (PIPAC #1 vs. PIPAC #2: *p* = 0.001; PIPAC #1 vs. PIPAC #3: *p* = 0.001; PIPAC #1 vs. PIPAC #4: *p* = 0.001). Postoperative CTCAE grade 4 complications were observed in two patients (6.9%) who had cytoreductive surgery (CC2) and intraoperative PIPAC. One patient (3.4%) died due to postoperative kidney insufficiency. After a follow up of 14.4 (95% CI: 8.1 to 20.7) months after the last PIPAC/PITAC application, median overall survival was 26.6 (95% CI: 9.5 to 43.7) months (from the first application).

**Conclusion:**

After prior abdominal surgery and systemic chemotherapy, repetitive PIPAC applications are feasible and safe for patients with end-stage MM. Furthermore, PIPAC induces significant histological regression of malignant mesothelioma in the majority of patients. PITAC is feasible, but its safety and efficacy to control malignant pleural effusion remain unclear.

**Electronic supplementary material:**

The online version of this article (10.1186/s12885-018-4363-0) contains supplementary material, which is available to authorized users.

## Background

Malignant epithelioid mesothelioma (MM) is a rare but aggressive malignancy arising from the mesothelial cells of the pleural cavity, peritoneal cavity, pericardium, or tunica vaginalis testis. MM account for less than 1% of all cancers [1]. Whereas malignant pleural mesotheliomas (MPM) are about two times more common than their peritoneal counterpart (MPeM), MMs of the pericardium and tunica vaginalis are extremely rare (1% - 2%) [2–6]. There is a clear association between the incidence of MPM and the degree of asbestos exposure and to a lesser extent also for MPeM and prior asbestos exposure [7–9]. Irrespective of its origin, with an observed median overall survival duration of 12 to 15 months, even in the area of modern multidisciplinary therapy approaches, the prognosis of MM remains poor [10, 11]. Mortality rates over the past 40 years have minimally decreased by 0.5% and 2% per year for MPM and MPeM, respectively [8]. In patients with MPeM who achieve complete or near complete surgical tumor resection and undergo combined heated intraperitoneal chemotherapy (HIPEC), estimated 5-year survival rates of 42% of patients can be achieved. Therefore, if feasible, this approach has become the standard of care for MPeM [12, 13].

Due to its nonspecific symptoms, MMs are often diagnosed late when disease burden is extensive and patients can only be managed with palliative chemotherapy. Since MPeM remains usually confined to the peritoneal cavity, loco-regional chemotherapy with direct exposure of the antitumor agent to the peritoneal tumor is considered an effective approach and overall tumor response rates for intraperitoneal chemotherapy (IPC) have been found to be higher compared to combined intravenous chemotherapy [14, 15].

Pressurized IntraPeritoneal Aerosol Chemotherapy (PIPAC) is a new technique to deliver intraperitoneal chemotherapy. During a standard laparoscopy, antitumor drugs are injected via a nebulizer into the abdominal cavity where a therapeutic aerosol is formed. Data from ex-vivo studies, animal experiments, and human studies demonstrated a higher local drug bioavailability and a better therapeutic index after PIPAC compared to liquid IPC [16]. Clinical safety, feasibility, and anti-tumor efficacy of PIPAC have been reported in ovarian and gastrointestinal cancer patients with peritoneal carcinomatosis [17]. The feasibility of Pressurized IntraThoracal Aerosol Chemotherapy (PITAC) to palliate malignant pleural effusion has been reported previously [18].

## Methods

### Patients and regulatory framework

Since April 2012, PIPAC/PITAC was applied with approved drugs for i.v. therapy as off-label use. Each patient was evaluated in a multidisciplinary tumor board. The indications for PIPAC were: i) medical co-morbidities and/or advanced disease excluding complete or near complete cytoreductive surgery and simultaneous heated intraperitoneal chemotherapy (CRS & HIPEC); ii) disease progression under/after systemic chemotherapy; iii) medical co-morbidities excluding systemic chemotherapy; iv) patients refusing CRS & HIPEC and/or systemic chemotherapy.

Pressurized IntraThoracal Aerosol Chemotherapy was administered in selected patients with clinically relevant malignant pleural effusion. PIPAC and PITAC were delivered simultaneously.

Patients with clinical signs of gastro-intestinal occlusion and/or a Karnofsky Index (KI) < 60% were excluded. We intended to deliver at least three PIPAC cycles separated by a six-week time interval. PITAC was only repeated in case of recurrence of significant pleural effusion or if a significant pleural effusion occurred on the contra-lateral side.

The study was performed in line with the guidelines of the Declaration of Helsinki and each patient was asked to give written informed consent for data collection as well as for publication of data in an anonymous manner. Data collection and analysis was done retrospectively within a prospective PIPAC registry approved by the local Institutional Review Board (Ethics Committee of the Ruhr-Universität Bochum, Germany; registration number 15–5280).

### PIPAC/PITAC procedure

The standard PIPAC procedure has been described in detail [16, 19]. The access to the abdominal cavity was obtained via a mini laparotomy lateral to the left rectus abdominus muscle in the midclavicular line at the level of the umbilicus. Peritoneal biopsies from all four abdominal quadrants (if possible) were retrieved and sent for histological analysis. Doxorubicin at a dose of 1.5 mg/m^2^ body surface in a total volume of 50 ml NaCl 0.9% followed by cisplatin at a dose of 7.5 mg/m^2^ body surface in a total volume of 150 ml NaCl 0.9% were aerosolized.

For PITAC, the patient was intubated with a double lumen endotracheal tube and then placed in a lateral thoracotomy position. After exclusion of the ipsilateral lung, access to the thoracic cavity was obtained via a 1-cm-incision in the 6th to 8th intercostal space in the mid axillary line and a 12-mm-trocar was inserted. The lung was then allowed to collapse and a second 5 mm trocar was placed under video-guidance. Pleural effusion was removed and multiple biopsies were taken. An intrathoracal pressure of 12 mmHg CO_2_ was established during the PITAC procedure. The technique, drugs and drug dosage were similar to PIPAC as described above. At the end of the procedure, a 12 Charrière chest tube was inserted and placed to a ventro-apical position and then connected to a digital chest drainage system with a continuous negative pressure of 15 cm H_2_O. With the patient still in the lateral position, the ipsilateral lung was then re-ventilated. In case of no air leak via the thoracic chest tube, the tube was then immediately removed. After extubation, a chest x-ray examination was performed. Senior surgeons trained in PIPAC/PITAC performed all procedures.

### Data collection, follow-up, and statistical analysis

Study nurses collected clinical data within a prospective PIPAC/PITAC registry and obtained follow-up data by telephone calls or questionnaires sent to family doctors/oncologist until the patient died or until the last follow-up (November 10, 2017). Quality of life was assessed by using EORTC QLQ-C30(+ 3) or EORTC QLQ-C30 (Version 3.0) questionnaires and combined/reported using the following scales: Global health status, QL2; physical functioning, PF or PF2; role functioning, RF2; all other scales are identical in the two versions of the questionnaires. Histological tumor response was assessed by the Institute of Pathology, Ruhr-Universität Bochum, Bochum, Germany. To evaluate the histological tumor regression grade (TRG) induced by PIPAC/PITAC, the following criteria according to Dworak et al. were applied: TRG 0 = no regression; TRG 1 = dominant tumor with obvious fibrosis with/without vasculopathy; TRG 2 = significant fibrotic changes with few tumor cells or groups (slightly recognizable histologically); TRG 3 = only scattered tumor cells in the space of fibrosis with/without acellular mucin; TGR 4 = no vital tumor cells detectable [20]. Whenever different scores were found in different tissue samples of the same patient, the lowest TRG value was reported. Adverse events were graded according to the Common Terminology Criteria for Adverse Events (v4.0 CTCAE) [21]. Data analysis was conducted retrospectively and is given as absolute numbers (N), per cent (%), mean (range: minimum to maximum) or median (confidence interval 95% (CI 95)). Overall median survival was modelled using a Kaplan-Meier curve. To compare independent samples, the Kruskal-Wallis test (ANOVA on ranks) was applied. Differences were considered significant at *p*-values < 0.05. Statistical analysis was performed with IBM SPSS Statistics 25.

## Results

### Patient characteristics

Between June 2012 and October 2017, a total of 29 patients (m/f = 17/12) with a mean age of 62.4 years (range: 42 to 84) and a mean Karnofsky Index of 85% (range: 60% to 100%) were included in the study. All patients had histologically proven peritoneal manifestations of MM. Histologically confirmed extra-abdominal manifestation of MM prior to the first PIPAC cycle was observed in thirteen patients (44.8%). Five patients (17.2%) were initially diagnosed with MPM but developed diaphragmatic disease extension into the peritoneal cavity during the later course of their disease. Two patients (6.9%) had initially undergone extrapleural pleuropneumonectomy and perioperative systemic chemotherapy but experienced thoracic recurrence.

With a mean number of 1.4 (range: zero to four) abdominal surgical interventions, ten patients (34.5%) had a total of fifteen extended abdominal surgical procedures with cytoreductive surgery (CRS) and hyperthermic intraperitoneal chemotherapy (HIPEC) or extended resection/debulking surgery without HIPEC. Six of those patients (20.7%) had undergone a total of seven CRS und HIPEC procedures. Another four patients (13.8%) had a total of eight extended resection/debulking surgery procedures. Resection/debulking surgery was performed due to initially suspected ovarian cancer in one and incidentally intraoperative discovered MPeM during elective colonic cancer surgery in another patient. Furthermore, two patients had a total of six major debulking interventions because of tumor progression with consecutive intestinal occlusion.

The mean number of prior systemic chemotherapy lines was 1.1 (range: zero to three). Eight patients (27.6%) had no complete line of systemic chemotherapy prior to PIPAC. The reasons for this were patient refusal or premature ending of systemic chemotherapy due to severe side effects in three patients each. Another two patients were referred to our peritoneal surface malignancies therapy center for suspected peritoneal metastasis of cancer of unknown origin in one case and in another case of ovarian origin. However, further work up revealed the diagnosis of MPeM. Both patients were not candidates for CRS and HIPEC so that both underwent first-line chemotherapy in combination with PIPAC. Tumor progress and/or tumor recurrence was documented in 21 patients (72.4%) before the first PIPAC/PITAC application. Seven patients (24.1%) received systemic chemotherapy combined with PIPAC treatment. The baseline demographic characteristics as well as details about prior therapies are summarized in Table 1.

**Table 1:** Baseline characteristics of patients scheduled for PIPAC/PITAC

Characteristics/Data items	Number	Percent
Total patients	29	100
Sex		
- Male	17	58.6
- Female	12	41.4
Mean age (years; range)	62.4 (42 to 84)	
ECOG performance score		
- 0 or 1	26	89.7
- 2 or 3	3	10.3
Karnofsky Index		
- 100%	2	6.9
- 90%	15	51.7
- 80%	9	31.0
- 70%	2	6.9
- 60%	1	3.4
Mesothelioma cell type		
- epithelioid	29	100
Clinical type		
- “dry” type	8	27.6
- “wet” type	12	41.4
- “mixed” type	9	31.0
Alcohol consumption		
- no or minimal	22	75.9
- daily alcohol intake	7	24.1
Smoking habits		
- no	18	62.0
- yes	11	38.0
Primary tumor site at initial diagnosis		
- abdominal	24	82.8
- thoracal	5	17.2
Prior extended resection surgery		
- cytoreduction & HIPEC (CC-0 & CC-1)	7	24.1
- abdominal debulking	8	27.6
- pleuropneumonectomy	2	6.9
- thoracotomy/decortication	3	10.3
Prior surgical score (PSS)		
- PSS 0	11	27.5
- PSS 1	8	29
- PSS 2	6	15
- PSS 3	15	37.5
Extra-abdominal metastatic spread before PIPAC/PITAC	13	44.8
- thoracal	8	27.6
- mediastinal	3	10.3
- abdominal wall	2	6.9
No previous systemic chemotherapy	8	27.6
- refused any systemic chemotherapy	3	10.3
- stop due to severe side effects	3	10.3
- new diagnosis of disease	2	6.9
First line chemotherapy	21	72.4
- Cisplatin & Pemetrexed	17	58.6
- Pemetrexed mono	3	10.3
- Carboplatin & Pemetrexed	1	3.4
Second line chemotherapy	8	27.6
- Cisplatin & Pemetrexed	5	17.2
- Pemetrexed mono	3	10.3
Third line chemotherapy	2	6.9
- Pemetrexed mono	2	6.9
Systemic chemotherapy & scheduled for PIPAC	7	24.1
- Cisplatin & Pemetrexed	4	13.8
- Pemetrexed mono	3	10.3
Scheduled for PIPAC without systemic chemotherapy	22	75.9

*HIPEC* hyperthermic intraperitoneal chemotherapy, *ECOG* Eastern Conference Oncology Study Group, *PIPAC* Pressurized IntraPeritoneal Aerosol Chemotherapy, *PITAC* Pressurized IntraThoracal Aerosol Chemotherapy, *PSS* prior surgical score, “wet” type = presence of ascites, “dry” = type absence of ascites

After a median time interval of 28 months (95% CI: 13 to 43) between the diagnosis of MM and the first PIPAC cycle, a total of 74 PIPAC procedures without any intraoperative complication were performed. The mean number of PIPAC applications was 2.5 (range: 0 to 10). Twelve of 29 patients (41.4%) underwent at least three PIPAC cycles.

### PIPAC applications

Minimal invasive access to the abdominal cavity for the first PIPAC application failed in seven patients (7/29), representing a primary non-access rate of 24.1%. However, in three of these patients, an open laparotomy was undertaken during a second surgical intervention. One patient had extensive adhesiolysis with subsequent PIPAC and two other patients had cytoreductive surgery (CC2) with simultaneous PIPAC. In the 25 patients (86%) who successfully received a first PIPAC cycle, a median PCI score of 19.9 (95% CI: 15.6 to 24.0) and malignant ascites of 1479 ml (95% CI: 703 to 2256) was documented. In the later course, in another three patients (3/29), minimal invasive access to the abdominal cavity was technically not feasible, representing a secondary non-access rate of 10%. Other reasons for premature ending of PIPAC applications (three cycles intended) were clinical deterioration in 5 cases, extra-abdominal tumor progression under PIPAC and palliative systemic chemotherapy in one case, patient decision to stop any therapy in two cases, no evidence of disease after tumor debulking and PIPAC in one case, and death in one case.

### Adverse events

With 34 postoperative complications (CTCAE grade 1–4) in a total of 79 PIPAC/PITAC applications, the procedure related overall morbidity rate (34/79) was 43.0%. Mild postoperative complications (CTCAE grade 1) such as abdominal pain, pronounced wound pain, nausea/vomiting, and temporary ascites leaking out of a 5 mm trocar incision occurred in 15% (12/79), 10% (8/79), 2% (2/79) and 1% (1/76), respectively. Transient prerenal kidney injury (CTCAE grade 2; creatinine 2.0–3.0 x above baseline) was observed after 8% (7/79) of interventions. A subcutaneous chemotherapy paravasation (CTCAE grade 3) after PIPAC therapy developed in one patient (1/79; 1.3%) at the 12 mm trocar entry site. A broad-spectrum antibiotic therapy, analgesia, and local antiphlogistic therapy were administered and the patient fully recovered. Severe CTCAE grade 4 complications due to postoperative small bowel anastomotic leakage was observed in two patients who had undergone subtotal cytoreductive surgery (CC2) and simultaneous PIPAC. However, both patients fully recovered postoperatively and are alive with a radiological stable disease and without systemic chemotherapy after a follow up period of 54 and 61 months, respectively.

The overall mortality rate of patients who had a minimum of one PIPAC application was 4% (1/25). The procedure-related mortality rate was 1.3% (1/79). One patient died after the second PIPAC cycle due to kidney insufficiency. Surgical details and postoperative adverse events are summarized in Table 2.

**Table 2:** Surgical details of PIPAC/PITAC

Data items	Number	Percent
Patients with primary non-access for PIPAC	7 / 29	24.1
Patients with secondary non-access for PIPAC	3 / 29	10.3
PIPAC procedures per patient (n)		
- 10 x	1 / 29	3.4
- 7 x	1 / 29	3.4
- 5 x	1 / 29	3.4
- 4 x	4 / 29	13.8
- 3 x	5 / 29	17.2
- 2 x	8 / 29	27.6
- 1 x	5 / 29	17.2
- 0 x	4 / 29	13.8
Number of successful PIPAC/PITAC procedures	79	100
- Laparoscopic PIPAC procedures	71 / 79	89.9
- Laparotomy, adhesiolysis +/− debulking & PIPAC	3 / 79	3.8
- PITAC procedures	5 / 79	6.3
Overall postoperative morbidity/mortality (CTCAE v 4.0)		
- Grade 1	23 / 79	29.1
- Grade 2	7 / 79	8.9
- Grade 3	1 / 79	1.3
- Grade 4	2 / 79	2.5
- Grade 5	1 / 79	1.3
Media survival time (days) according to tumor regression grade		
- Grade 0	6	226 (31–708)
- Grade 1	11	360 (135–529)
- Grade 2+	8	360 (175–1331)

*PIPAC* Pressurized IntraPeritoneal Aerosol Chemotherapy, *PITAC* Pressurized IntraThoracal Aerosol Chemotherapy

### Tumor regression assessment, Ascites & Survival

**Fig. 1:**
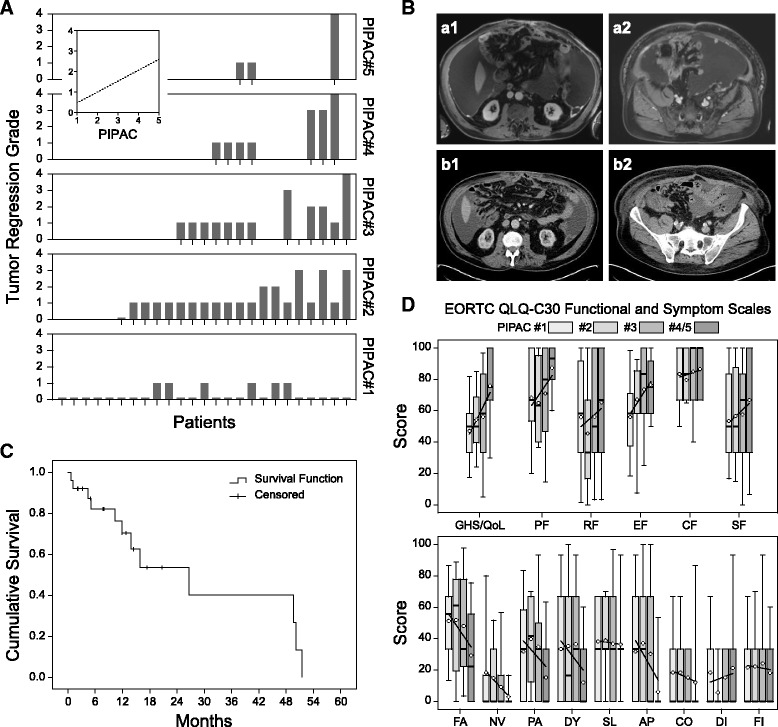
**a** Tumor regression induced by PIPAC treatments. Bars indicate tumor regression in individual patients undergoing PIPAC cycles 1 to 5. Inset with dotted line: linear regression (*p* < 0.001; regression coefficient = 0.587). Tumor regression grade 0 to 4 according to Dworak et al.: 0, no regression; 1, minimal regression; 2, moderate regression; 3, major regression; 4, complete regression. **b** MRI and CT scans of a 59 year old patient who refused any systemic chemotherapy and not being a candidate for cytoreductive surgery and HIPEC. Upper panel: Preoperative MRI before PIPAC#1 showing the upper abdomen with pronounced ascites perihepatic, in the left upper abdomen (a1) and in the lower abdomen/pelvis with a trapped small intestine (a2). During PIPAC#1, 6 l of ascites were evacuated. Lower panel: CT scan of the upper abdomen 12 weeks after PIPAC#1 (6 weeks after PIPAC#2) and preoperatively before PIPAC #3, showing only a moderate amount of ascites perihepatic, in the left upper abdomen (b1) and pelvis (b2). 500 ml ascites was removed during PIPAC#3. **c** In 25 patients who received at least one PIPAC cycle, a median overall survival of 26.6 months (95% CI: 9.5 to 43.7) was observed after the first PIPAC application (open and laparoscopic). **d** Quality of life scores according to the EORTC QLQ-C30 questionnaire during PIPAC treatment cycles 1 (*N* = 21), 2 (*N* = 18), 3 (*N* = 11), and 4/5 (*N* = 11), respectively. Box plots: lower/upper boundaries of boxes represent the 25th/75th percentiles, thick lines the medians, and the whiskers the 10th and 90th percentiles, respectively; white circles are the means (with regression lines). GHS/QoL, global health score, quality of life; PF, physical functioning; RF, role functioning; EF, emotional functioning; CF, cognitive functioning; SF, social functioning; FA, fatigue; NV, nausea and vomiting; PA, pain; DY, dyspnoea; SL, insomnia; AP, appetite loss; CO, constipation; DI, diarrhoea; FI, financial difficulties

The median number of biopsy samples harvested during all 74 PIPACs was 8 (95% CI: 6 to 12) per patient. A local peritonectomy specimen of 3 × 3 cm was obtained in every case. From 25 patients, tumor samples were retrieved during the staging laparoscopy prior to the first PIPAC. No tumor regression (TRG 0) was observed in 19 patients (19/25; 76%). However, in six patients (6/25; 24%), minimal tumor regression (TRG 1) was found. All of these patients had previous systemic chemotherapy. Out of a total of 29 patients, 20 patients (69%) underwent ≥2 PIPAC cycles and were therefore eligible for histological tumor regression grade (TRG) analysis. Repetitive PIPAC applications failed to induce any objective histological tumor regression in 25% of patients (5/20). However, 75% of patients (15/20) showed an objective tumor regression with major (TRG 3) and complete tumor regression (TRG 4) observed in 20% (4/20) and 10% (2/20), respectively. On an intention-to-treat basis, PIPAC thus caused histological tumor regression in 51.7% of patients (15/29). Comparison of TRG scores between consecutive PIPAC cycles showed a significant increase in TRG after the first PIPAC but no further significant increase for the following PIPAC applications (PIPAC #1 vs. PIPAC #2: *p < 0.0001*; PIPAC #1 vs. PIPAC #3: *p < 0.0001*; PIPAC #1 vs. PIPAC #4: *p* < *0.0001).* There was a significant linear regression (*p < 0.001*; regression coefficient = 0.587). Figure 1a summarises the histological tumor regression grade (TRG) induced by PIPAC treatment.

Although there is a trend towards a decrease of malignant ascites under repetitive PIPAC applications, no statistically significant effect was observed (*p = 0.99*). In some patients, PIPAC induced a marked reduction of malignant ascites. Figure 1b shows a follow-up computer tomography (CT) after PIPAC application. Similar results were observed for the PCI score, which did not change significantly during PIPAC therapy (*p > 0.99*). Consecutive ascites volume measurements as well as the PCI score assessments during PIPAC treatment did not show any significant changes (Additional file 1: Figure S1 and Additional file 2: Figure S2).

The follow up period started with the first PIPAC application. During a median follow up period of 14.4 months (95% CI: 8.1 to 20.7), a median overall survival of 26.6 months (95% CI: 9.5 to 43.7) was observed. At the end of the study period, a total of ten patients had died. Survival data of 25 patients after the first PIPAC cycle are given in Fig. 1c.

Quality of life was assessed and demonstrated a notable increase in all functional scales such as physical functioning, role functioning, emotional functioning, social functioning, and cognitive functioning as well as overall quality of life (Fig. 1d ). In addition, items of gastrointestinal toxicity such as appetite loss, constipation, nausea, and emesis also improved during PIPAC treatment as did fatigue (Fig. 1d).

### PITAC applications

Three patients underwent a total of five PITAC procedures in addition to PIPAC during the same operation. PITACs were repeated within an interval of six weeks for recurrence of significant malignant pleural effusion. The mean amount of pleural effusion evacuated during the first PITAC was 1150 ml (range: 900 to 1900). No perioperative complications occurred. In one patient, recurrence of a clinical relevant pleural effusion was observed four weeks after the first PITAC procedure together with a rapid decline of the patient’s general condition. Therefore, no further PIPAC/PITAC procedure was performed and the patient died 63 days after the first PIPAC/PITAC with best supportive care. In the two other patients, follow-up CT scans six months after the first PITAC/PITAC showed a stable condition with a moderate amount of pleural effusion of 200 ml and 300 ml, respectively.

## Discussion

In Europe, the incidence of MM is increasing with an expected peak incidence between the years 2010 and 2040 [10]. Moreover, the prognosis of this disease remains poor for most patients. However, patients with MM of the abdominal cavity who undergo macroscopic complete tumor resection and perioperative intraperitoneal chemotherapy, 5-year survival rates of 30% to 60% have been reported by several independent observational studies [22]. Unfortunately, most patients are not suitable for extensive surgery and therefore are treated with systemic palliative chemotherapy with a median overall survival of 15 months [11]. Since MPeM usually remains confined to the abdominal cavity for most of its natural history, this biological behavior predisposes these patients to benefit from intraperitoneal therapy [8].

PIPAC is a new and optimized intraperitoneal chemotherapy approach for patients with end-stage peritoneal carcinomatosis. In contrast to conventional liquid intraperitoneal chemotherapy, PIPAC delivers cytostatic drugs into the abdominal cavity as a therapeutic aerosol during a standard laparoscopy. The rationale behind this approach is based on the facts that pressure enhances the in-tissue drug influx by convection [23], an aerosol has a superior surface/volume ratio compared to liquids, and the higher drug concentration of the therapeutic aerosol increases the in-tissue depth penetration and concentration by enhanced diffusion [24]. In addition, PIPAC has the advantage that it can be given repetitively and the therapy response can be monitored objectively by repetitive tumor biopsies. Published data on the safety and feasibility from our PIPAC program as well as data from independent groups confirm a high feasibility and safety profile of PIPAC treatment in ovarian, gastric, colo-rectal an pancreatic cancer patients [17, 25].

In the present series, primary non-access was observed in 24% of patients but no intraoperative complications occurred. However, due to a lack of alternative therapy options, we performed laparotomy, adhesiolysis, even cytoreductive surgery (CC2) with simultaneous or consecutive PIPAC in these patients. Within our series, the primary non-access rate was somewhat higher than that observed in previous reports [25, 26] but still lower than recently published by a French multicenter trial that found a high non-access rate of 38% [27]. Furthermore, postoperative complications after PIPAC applications were frequently observed but were within the range of other published PIPAC series [17]. However, in our series, two patients with cytoreductive surgery and simultaneous PIPAC application had postoperative small bowel anastomotic leakage that required emergency surgery (CTCAE grade 4), multiple reoperations and prolonged postoperative recovery. Although this observation might only be a coincidence, it is most likely to be linked to the fact that tissue concentrations after PIPAC application are far higher compared to those observed for HIPEC [16]. Moreover, ex-vivo studies report, that the highest tissue in-depth chemotherapy penetration occurs in the small bowel [28, 29]. Based on these observations and preclinical pharmacological data we do not recommend complex adhesiolysis, cytoreductive surgery and simultaneous PIPAC.

PIPAC- and HIPEC-related mortality between 1% and 3% is well documented in experienced centers using these techniques [17]. However, also in this present study, one patient died after PIPAC application. The patient presented intraoperatively with extensive and bulky tumor manifestations and developed the typical metabolic findings of tumor lysis syndrome after PIPAC and died due to acute kidney failure.

To date, few studies have been reported of patients with MPeM, and treatment of this disease has been largely extrapolated from the treatment of MPM. However, recent data about cisplatin combined with pemetrexed demonstrated improved disease control rates [11, 30]. Fujimoto et al. treated twenty-four patients with histologically proven MPeM with cisplatin and pemetrexed as first-line therapy. A total of thirteen patients were eligible for metabolic tumor response analysis by ^18^F-fluorodeoxyglucose positron emission tomography (FDG/PET). An overall response rate of 46% and a median overall survival of 16 months were observed [11].

PIPAC offers the unique opportunity of objectively analyzing and monitoring tumor regression during PIPAC. Overall, in our population of extensively pretreated patients, PIPAC induced objective tumor regression > 50% of patients whereas major and even complete tumor response was achieved in 20% of patients. Comparing these findings with the results of first-line systemic chemotherapy with cisplatin and pemetrexed reported by Fujimoto et al. suggests that PIPAC might be a suitable second-line therapy after failure of standard first-line therapy with surgery and a cisplatin/pemetrexed systemic chemotherapy regimen. Although the observed survival rate and duration is promising, it remains unclear whether this effect is attributed to PIPAC or to selection bias. For example, almost half of the patients presented clinically as “wet”-type mesothelioma. It has been reported recently that this MM subtype has a prolonged overall survival of forty-one months with first-line cisplatin and pemetrexed [11].

A further positive aspect is the general improvement in the quality of life reported by patients undergoing PIPAC. Quality of life assessments demonstrated a notable increase in all functional scales such as physical functioning, role functioning, emotional functioning, social functioning, and cognitive functioning as well as overall quality of life. In addition, items of gastrointestinal toxicity such as appetite loss, constipation, nausea, and emesis also improved during PIPAC treatment as did fatigue. This is a notably finding and underscores the potential validity of PIPAC in patients with MM.

We recommend not performing adhesiolysis when accessing the abdomen during PIPAC. PIPAC is an aggressive local therapy and small serosal lesions occurring during adhesiolysis may put the bowel wall at risk for disintegration when exposed to the chemotherapy compounds applied during PIPAC. This is, however, an empirical recommendation based on the observation that chemical bowel perforation did not occur in our patients, who generally did not undergo adhesiolysis.

Clearly, the sample size is a limitation of our study and has to be kept in mind when interpreting the study results. On the other hand, MM is a rare disease and case series reporting on patients with MM are usually limited in size. In addition, PIPAC is a new procedure and our results have to be independently confirmed. Lastly, the retrospective design of this study limits the generalizability of the results. Selection of patients for this procedure as well as self selection may influence the results favorably for PIPAC. Future studies should thus be prospective and implement uniform inclusion criteria.

## Conclusions

Delivering repetitive PIPAC cycles to patients with end-stage MM of the abdominal cavity who had previous major abdominal surgery and systemic chemotherapy is feasible and safe. MM of the peritoneal cavity is highly sensitive to PIPAC achieving significant tumor regression. The median overall survival time of 26.6 months is promising. PITAC is feasible but its safety and efficacy to control malignant pleural effusion remain unclear.

## Additional files

Additional file 1: **Figure S1.** Box plots of malignant ascites removed during PIPAC cycles #1 to #5. No statistically significant control of malignant ascites could be achieved with PIPAC treatment (*p* > 0.99). (PDF 101 kb)

Additional file 2: **Figure S2.** Box plots for the PCI observed during PIPAC cycles #1 to #5. No statistically significant difference could be observed during repetitive PIPAC applications (*p* > 0.99). (PDF 22 kb)

## Abbreviations

CC: Completeness of cytoreduction
CI: Confidence interval
CTCEA: Common Terminology Criteria for Adverse Events v4.0
ECOG: Eastern Cooperative Oncology Group Performance Score
IPC: Intraperitoneal chemotherapy
KI: Karnofsky index
MM: Malignant mesothelioma
MPeM: Malignant peritoneal mesothelioma
MPM: Malignant pleural mesothelioma
PC: Peritoneal carcinomatosis
PCI: Sugarbaker’s peritoneal carcinomatosis index
PIPAC: Pressurized IntraPeritoneal Aerosol Chemotherapy
PITAC: Pressurized IntraThoracal Aerosol Chemotherapy
PSS: Prior surgical score
TRG: Tumor regression grade
