# Supplementary figures and images for: Pressurized IntraPeritoneal Aerosol Chemotherapy (PIPAC) for the treatment of malignant mesothelioma
Source: BMC Cancer. 2018 Apr 18;18:442. doi: 10.1186/s12885-018-4363-0 (PMC5907219; doi:10.1186/s12885-018-4363-0)

Supplementary Figure S1

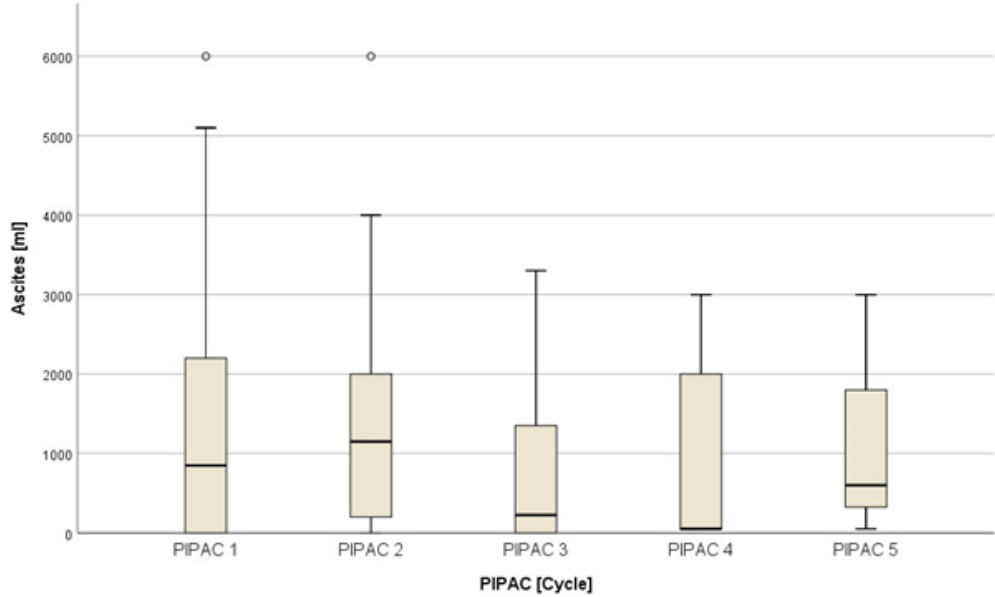

Supplement: Supplementary file 1 — Figure S1. Box plots of malignant ascites removed during PIPAC cycles #1 to #5. No statistically significant control of malignant ascites could be achieved with PIPAC treatment (p > 0.99). (PDF 101 kb) [file 12885_2018_4363_MOESM1_ESM.pdf]

Supplementary Figure S2

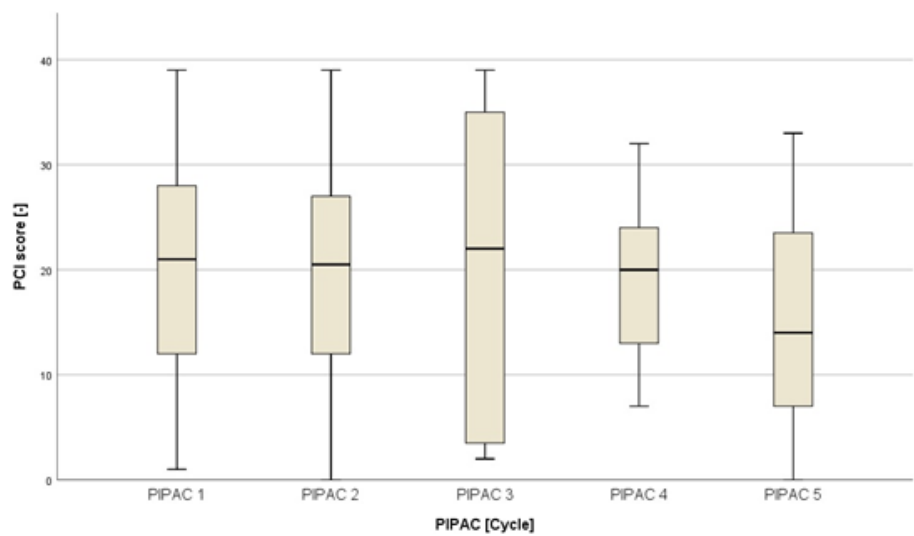

Supplement: Supplementary file 2 — Figure S2. Box plots for the PCI observed during PIPAC cycles #1 to #5. No statistically significant difference could be observed during repetitive PIPAC applications (p > 0.99). (PDF 22 kb) [file 12885_2018_4363_MOESM2_ESM.pdf]
